# Chronic AMPK activity dysregulation produces myocardial insulin resistance in the human Arg302Gln-PRKAG2 glycogen storage disease mouse model

**DOI:** 10.1186/2191-219X-3-48

**Published:** 2013-07-05

**Authors:** Stephanie L Thorn, Michael H Gollob, Mary-Ellen Harper, Rob S Beanlands, Robert A deKemp, Jean N DaSilva

**Affiliations:** 1 National Cardiac PET Centre, Division of Cardiology, University of Ottawa Heart Institute, 40 Ruskin St, Ottawa, Ontario K1Y 4W7, Canada; 2 Department of Cellular and Molecular Medicine, Faculty of Medicine, University of Ottawa, 451 Smyth Rd, Ottawa, Ontario K1H 8M5, Canada; 3 Department of Biochemistry, Microbiology, and Immunology, Faculty of Medicine, University of Ottawa, 451 Smyth Rd, Ottawa, Ontario K1H 8M5, Canada

**Keywords:** Positron emission tomography, FDG, Patlak, Echocardiography, Glucose

## Abstract

**Background:**

The cardiac PRKAG2 mutation in the γ2-subunit of adenosine monophosphate activated kinase (AMPK) is characterized by excessive glycogen deposition, hypertrophy, frequent arrhythmias, and progressive conduction system disease. We investigated whether myocardial glucose uptake (MGU) was augmented following insulin stimulation in a mouse model of the PRKAG2 cardiac syndrome.

**Methods:**

Myocardial and skeletal muscle glucose uptake was assessed with 2-[^18^F]fluoro-2-deoxyglucose positron emission tomography imaging in *n* = 3 transgenic wildtype (TGwt) vs *n* = 7 PRKAG2 mutant (TGmut) mice at baseline and 1 week later, 30 min following acute insulin. Systolic function, cardiac glycogen stores, phospho-AMPK α, and insulin-receptor expression levels were analyzed to corroborate to the *in vivo* findings.

**Results:**

TGmut Patlak Ki was reduced 56% at baseline compared to TGwt (0.3 ± 0.2 vs 0.7 ± 0.1, *t* test *p* = 0.01). MGU was augmented 71% in TGwt mice following acute insulin from baseline (0.7 ± 0.1 to 1.2 ± 0.2, *t* test *p* < 0.05). No change was observed in TGmut mice. As expected for this cardiac specific transgene, skeletal muscle was unaffected at baseline with a 33% to 38% increase (standard uptake values) for both genotypes following insulin stimulation. TGmut mice had a 47% reduction in systolic function with a fourfold increase in cardiac glycogen stores correlated with a 29% reduction in phospho-AMPK α levels. There was no difference in cardiac insulin receptor expression between mouse genotypes.

**Conclusions:**

These results demonstrate a correlation between insulin resistance and AMPK activity and provide the basis for the use of this animal model for assessing metabolic therapy in the treatment of affected PRKAG2 patients.

## Background

Insulin resistance is strongly associated with the development of heart failure due to cardiotoxicity induced by the hyperglycemic state with subsequent modification of contractile proteins [1] and development of cardiac dysfunction [2]. The potential to decrease insulin resistance and increase glucose uptake has recently been sought through the upregulation of adenosine monophosphate activated kinase (AMPK) activity [3]. AMPK acts as a cellular fuel gauge, tightly regulating glucose and fatty acid metabolism. Indeed, metformin, a common pharmacological treatment for type 2 diabetes, targets AMPK subsequently, increasing insulin sensitivity [4-6].

The critical importance of AMPK function in the heart was highlighted by the observation that genetic mutations in the *PRKAG2* gene, encoding the gamma-2 regulatory subunit of AMPK, gives rise to a novel cardiac glycogenosis syndrome in humans [7,8]. Affected individuals exhibit varying degrees of cardiac hypertrophy, frequent and persistent cardiac arrhythmias, and progressive conduction system disease leading to pacemaker implantation. In some cases, the need for heart transplantation or premature sudden death may occur [9-11]. On gross pathologic and histologic assessment, the hallmark of the PRKAG2 cardiac syndrome is the diffuse and extensive glycogen deposition throughout all four cardiac chambers [12].

Using 2-[^18^F]fluoro-2-deoxyglucose (FDG) and positron emission tomography (PET) imaging, human patients with the Arg302Gln mutation of PRKAG2 exhibited a 45% reduction in myocardial glucose uptake compared to control subjects [13].

This study sought to evaluate whether the PRKAG2 myocardium is responsive to insulin-mediated cardiac glucose uptake. To address this, a transgenic mouse model of the human PRKAG2 cardiac syndrome was assessed with *in vivo* FDG PET imaging at baseline and 1 week later following insulin stimulation. We further evaluated whether this response correlated to changes in protein expression of activated AMPK, insulin receptors, and cardiac glycogen content.

## Methods

### Animal model

All animal experiments described herein were conducted according to the guidelines of the University of Ottawa Animal Care Committee and the Canadian Council on Animal Care for the use and care of laboratory animals. All animals were maintained on a 12-h light/dark cycle with chow (Harlan Tekland #2019) and water *ad libitum*.

Transgenic mice were generated as previously described [14] using a cardiac-specific α-myosin heavy chain promoter, PRKAG2 cDNA, 3′UTR human growth hormone. These mice, therefore, have the transgene only in the myocardium. Transgenic mutant (TGmut) mice express the mutant PRKAG2 gene (substitute of arginine for glutamine at residue 302), with transgenic wildtype (TGwt) mice expressing the normal human PRKAG2 complementary deoxyribonucleic acid (cDNA). PCR was performed on the genomic DNA isolated from weanling mouse tail snips with the QIAGEN DNeasy kit (Venlo, Netherlands) with the product sequenced. All studies herein were performed in mice at 5 to 8 months of age (TGwt *n* = 7, TGmut *n* = 11).

### Echocardiography

Echocardiography images were obtained with the Vevo 770 imaging system (VisualSonics, Toronto, ON, Canada) using a 707 transducer. Mice (TGwt *n* = 4, TGmut *n* = 4) were anesthetized and maintained throughout experimental procedures with 1% to 2% isoflurane. Long-axis B-mode images were assessed with the commercially available VisualSonics cardiac measurements program to determine left ventricle (LV) fractional shortening and endocardial volume.

### Small animal PET imaging

Small animal PET FDG imaging was conducted with the Inveon^TM^ DPET small animal scanner (Siemens, Knoxville, TN, USA). Mice (TGwt *n* = 3, TGmut *n* = 7) were scanned under anesthetic (1% to 2% isoflurane, 2 to 2.5 mL/min oxygen) at baseline and 1 week later, 30 min following an acute intraperitoneal injection with short-acting insulin (8 mU/g body weight; Novolin ge Toronto, Novo Nordisk, Denmark) [15]. A 60-min list-mode acquisition was started together with a 10- to 20-s tail vein injection of FDG (25.3 ± 7.9 MBq in 150 μL). List data were sorted into 26 dynamic frames (12 × 10, 3 × 60, 11 × 300 s) and reconstructed using OSEM3D with 10 iterations, 16 subsets, and zoom of 2.5 with a 128 × 128 matrix, resulting in 0.35 mm transaxial pixel size. Images were corrected for radioactive decay, random coincidences, and dead-time losses using the vendor software (IAW version 1.5). Blood glucose concentration was measured (mmol/L) prior to FDG injection with a small drop of blood from the saphenous vein using Advantage blood glucose strips (AccuChek, Roche Diagnostics, Laval, QC, Canada).

### Image analysis

Myocardial uptake images of the LV were formed by averaging the last 5 min of scan data using FlowQuant© semi-automated software [16]. The location, orientation, and size of the LV were automatically determined by fitting ellipses to the myocardium in the transverse, vertical long axis and horizontal long axis planes. Transverse uptake images were reoriented automatically into short-axis (SA) sections generating LV slices from the apex to the base plane. Polar maps of the relative uptake activity (%) were formed from the sampled data. The sampling points were then applied to all-time frames to generate myocardial time-activity curves (TACs). Myocardial TACs were compared using standard uptake values (SUV) calculated by dividing the activity concentration in the region of interest (Bq/cc) by the activity concentration per body weight injected into the animal (Bq/g). Skeletal muscle SUV values (Siemens IRW, Munich, Germany) were also evaluated at 7.5 and 60 min at both baseline and following insulin as a control region of interest for the cardiac genetic phenotype. Blood regions of interest were derived with the Siemens IRW software using the proximal vena cava and the resulting blood TACs were imported into FlowQuant© image analysis software as previously described [16]. Patlak kinetic analysis [17] was performed on the myocardium at 1.58 to 7.5 min from the reconstructed images (FlowQuant©). The relationship after the initial input function between the myocardial activity (C_m_[t]) corrected for blood activity (Cb[t]) and the integral of blood activity (C_b_[t]) at time (t) becomes linear, with the slope representative of the uptake rate constant Ki.

### Protein expression and cardiac glycogen levels

Mice (TGwt *n* = 4, TGmut *n* = 4) were euthanized by decapitation with hearts dissected out, then placed immediately into liquid nitrogen for storage at −80°C. Tissue was ground using a mortar and pestle under liquid nitrogen. Glycogen content was determined using an amyloglucosidase digestion method as previously described [18]. Briefly, 10 to 50 mg of ground cardiac tissue was homogenized in a 1:9 (*wt./vol.*) ratio of PBM buffer (20 mM KH_2_PO_4_, 10 μM CaCl_2_, 1 mM MgCl_2_, pH 6.1). Tissue homogenate (50 μL) was boiled for 20 min in 30% KOH (*wt./vol.*) saturated with anhydrous Na_2_SO_4_. The remainder of tissue homogenate was used to determine protein concentration with a bicinchoninic acid (BCA) assay. Glycogen was precipitated with 95% ethanol (*vol./vol.*), centrifuged at 4,000 × *g* for 15 min at 4°C. The glycogen pellet was dissolved in double distilled H_2_O and incubated at 100°C for 20 min with 0.2% anthrone (*wt./vol.*) in H_2_SO_4_. Absorbance was read at 595 nm to determine glycogen concentration of samples relative to an oyster glycogen standard curve (Sigma Aldrich, Canada) and normalized to protein content (microgram of glycogen per microgram of protein). Ground tissue was homogenized in buffer (10 mM Tris–HCl, 50 mM NaF, 1 mM EDTA, 10 mM dithiothreitol, 10% glycerol (*vol./vol.*), pH 7.5 at 4°C) and a BCA assay performed to determine protein concentration. Tissue lysate (20 μg) was applied to each lane of an 8% SDS-polyacrylamide gel with separated bands of proteins transferred to a polyvinylidene fluoride membrane. Blocked membranes were incubated with either AMPK α (1:1,000), phosphorylated-AMPK α (Thr 172) (1:500), or insulin receptor β (1:1,000) (Cell Signaling Technology, Danvers, MA, USA). A mouse glyceraldehyde 3-phosphate dehydrogenase (GAPDH) antibody (Santa Cruz Biotechnology, Inc, Santa Cruz, CA, USA) was used as a loading control. Membranes were analyzed using a Fluor Chem HD image system (Alpha Innotech, San Leandro, CA, USA). Data were expressed as a ratio of target protein to GAPDH protein band intensities, normalized to the mean of TGwt controls for each membrane.

### Statistical analysis

Curve fitting with FlowQuant© software was performed using MATLAB (The MathWorks, Natick, MA, USA). All data are expressed as mean ± standard deviation. Data were compared using a Student's *t* test or a one-way ANOVA with Bonferroni correction; *p* < 0.05 was considered significant.

## Results and discussion

### Results

#### Echocardiography

**Figure 1:**
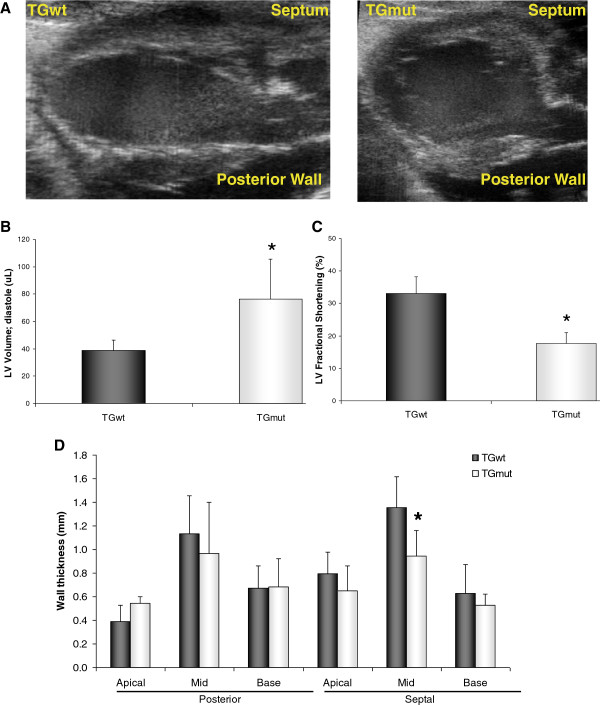
**Arg302Gln PRKAG2 increases LV volume and reduces cardiac function.** Representative echocardiography M-mode images **(A)** of a TGwt and TGmut mouse. Using B-mode echocardiography measurements of the long axis, comparison of LV volume **(B)**, fractional shortening **(C)**, and wall thickness **(D)**. TGwt *n* = 4 and TGmut *n* = 4. *p < 0.05, Student's *t* test.

Cardiac function, volume, and wall thickness were assessed with echocardiography *in vivo*. Examples of long-axis B-mode (Figure 1A) images are displayed for a TGwt and TGmut mouse. The Arg302Gln PRKAG2 TGmut mice have a 96% increase in the LV volume compared to TGwt controls (76.3 ± 29.3 μL vs 38.9 ± 7.3 μL; *t* test *p* < 0.05) (Figure 1B). Additionally, these mice display an overall reduction in cardiac systolic function with LV fractional shortening reduced to 47% (TGwt 33.1% ± 5.1% vs TGmut 17.7% ± 3.3%; *t* test *p* < 0.01) (Figure 1C) with similarity in heart rates between groups (TGwt 407 ± 35 bpm, TGmut 360 ± 69 bpm; *p* = 0.27). There was no difference in LV wall thickness in TGmut except for 30% significant reduction in the mid septal wall (1.4 ± 0.3 vs 0.9 ± 0.2; *t* test *p* < 0.05) (Figure 1D).

#### Myocardial glucose uptake is reduced in TGmut mice with no response to insulin stimulation

**Figure 2:**
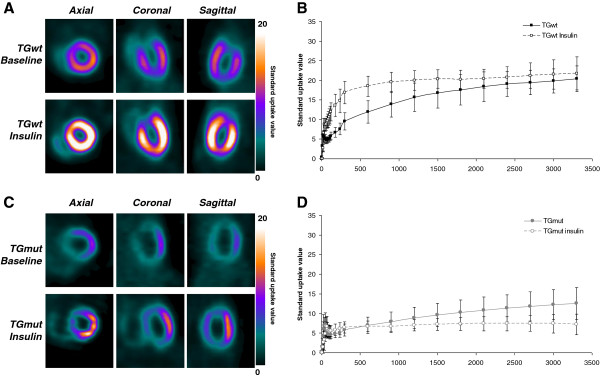
**Representative microPET FDG images and SUV time-activity curves at baseline and following acute insulin treatment.** Representative cardiac FDG images of TGwt **(A)** and TGmut mice **(C)** at baseline and following insulin stimulation. Images are presented in the axial, coronal, and sagittal views and standardized to the same scale. Myocardial TAC baseline and following insulin in TGwt **(B)** and TGmut mice **(D)**, respectively, over 60 min using SUV. TGwt *n* = 3 and TGmut *n* = 7.

**Figure 3:**
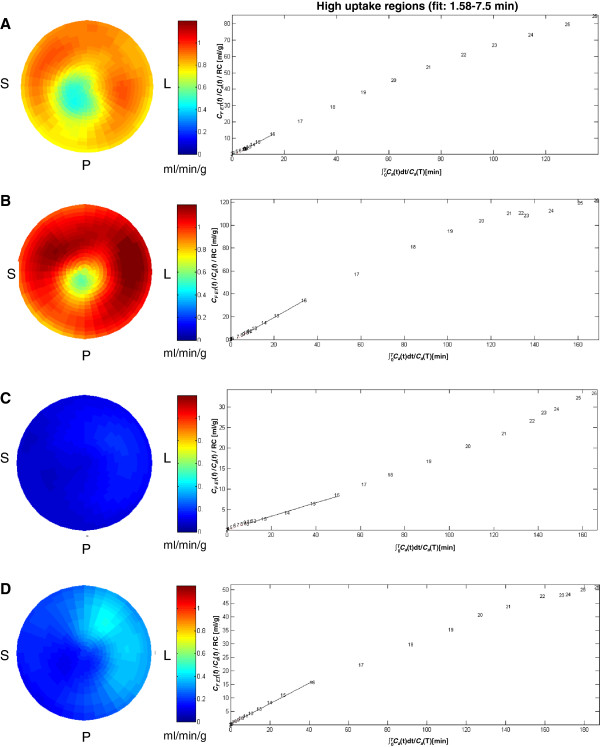
**Representative FDG Patlak Ki polar maps and linear plots.** FDG Patlak Ki polar maps for TGwt baseline **(A)**, TGwt with insulin **(B)**, TGmut baseline **(C)**, and TGmut following insulin **(D)**. Representative mean derivation of Patlak analysis showing a linear plot of normalized activity vs normalized time are shown beside the polar map for each state. Solid lines represent the fit of the Patlak Ki to the linear curves at 1.58 to 7.5 min. The slope of this relationship quantifies Patlak Ki. TGwt *n* = 3 and TGmut *n* = 6.

There was no difference between baseline and follow-up scans or between genotype with respect to body weight at time of scan (*p* > 0.05 one-way ANOVA, Bonferrroni; Table 1). Blood glucose levels were not significantly different between TGwt and TGmut mice at baseline (Table 1), both groups showing a similar decrease of 60% to 70% in blood glucose values following insulin pretreatment prior to FDG injection (Table 1). Representative cardiac images in TGwt (Figure 2A) and TGmut (Figure 2C) are depicted in axial, coronal, and sagittal views at baseline and following insulin stimulation. Contrary to TGmut mice, SUV analysis of the myocardial curves displays an increase in initial uptake rate with insulin stimulation in TGwt mice (Figure 2B, D). In fact, a 43% reduction in myocardial glucose uptake following acute insulin stimulation at 60 min was observed in TGmut mice (Table 2). Both TGwt and TGmut show a 31% to 38% increase in skeletal muscle SUV values with insulin stimulation at both 7.5 and 60 min (Table 2). Patlak kinetic analysis was used to determine differences in myocardial glucose uptake rate. Representative Patlak Ki polar maps and linear plots of normalized activity in high-uptake regions between 1.58 and 7.5 min are displayed (Figure 3). Patlak polar maps qualitatively show an increase in Ki values with insulin in TGwt mice (Figure 3A, B) but not observed in the TGmut mice (Figure 3C, D). In all cases, there was an excellent linear fit in the time frame of 1.5 to 7.5 min. At baseline, Patlak Ki was reduced 56% in TGmut mouse hearts compared to TGwt mice (Table 3). The TGwt display an increase of 71% from baseline following insulin stimulation while TGmut showed no change in cardiac uptake rates following insulin stimulation (Table 3).

**Table 1:** Body weight and blood glucose values prior to injection of FDG

**Mouse Genotype**	**Body weight (g)**	**Pre-FDG blood glucose (mmol/L)**
TGwt	36.4 ± 8.3	14.1 ± 1.3
TGwt insulin	37.7 ± 8.9	4.0 ± 0.3*
TGmut	32.2 ± 4.5	10.4 ± 3.4
TGmut insulin	33.2 ± 4.1	4.6 ± 1.9*

*p < 0.05 by ANOVA and after Bonferroni correction.

**Table 2:** Myocardium and skeletal muscle FDG standard uptake values (SUV)

**SUV**	**Myocardium**	**Muscle**
SUV 7.5 min p.i.		
TGwt	9.6 ± 2.2	0.5 ± 0.1
TGwt insulin	16.9 ± 2.8*	0.8 ± 0.2*
TGmut	5.8 ± 1.6**	0.7 ± 0.1
TGmut insulin	6.4 ± 1.4	1.1 ± 0.5*
SUV 60 min p.i.		
TGwt	20.4 ± 3.3	0.7 ± 0.2
TGwt insulin	21.8 ± 4.3	1.1 ± 0.2*
TGmut	12.6 ± 4.1	1.0 ± 0.2
TGmut insulin	7.2 ± 2.6*	1.5 ± 0.6*

p.i., post injection; *p < 0.05 vs non-insulin treated baseline with Student's *t* test; **p < 0.05 vs TGwt with Student's *t* test.

**Table 3:** Myocardial FDG Patlak kinetic analysis (1.58 to 7.5 min)

**Mouse Genotype**	**Baseline**	**Insulin**
TGwt	0.7 ± 0.1	1.2 ± 0.1*
TGmut	0.3 ± 0.2**	0.4 ± 0.2

*p < 0.05 vs non-insulin-treated baseline with Student's *t* test; **p < 0.05 vs TGwt with Student's *t* test.

#### Glycogen deposition and protein expression

**Figure 4:**
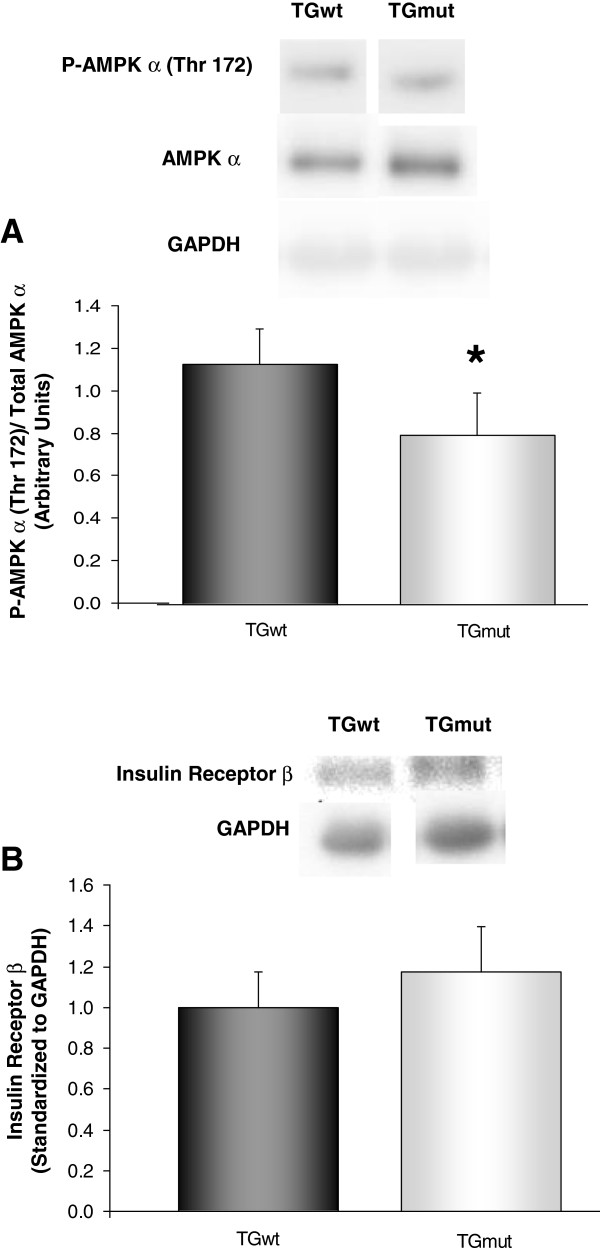
**Protein expression of AMPK and insulin receptor β.** Relative protein expression normalized by measured GAPDH levels and then normalized to TGwt for phospho-AMPK α levels expressed to total AMPK **(A)** and insulin-receptor protein expression **(B)**. TGwt *n* = 4 and TGmut *n* = 4; *p < 0.05, Student's *t* test.

There was a fourfold increase in cardiac glycogen levels in the TGmut mice compared to TGwt hearts (0.46 ± 0.07 vs 0.11 ± 0.05; *t* test *p* < 0.001). A 29% reduction in phosphorylation of AMPK α on Thr 172 normalized to total AMPK expression levels was found in TGmut mice compared to TGwt hearts (1.1 ± 0.2 vs 0.8 ± 0.2; *t* test *p* < 0.05) (Figure 4A). Protein expression of cardiac insulin receptor β was not affected by the PRKAG2 genotype with no difference found between groups (Figure 4B).

## Discussion

Central to cardiac metabolism, AMPK is activated in response to energy deprivation to rapidly modulate cardiac metabolism [19]. Activated AMPK is known to enhance translocation of GLUT4 to the cell membrane to facilitate glucose uptake [20]. In the current study, we evaluated mice at 5 to 8 months of age and demonstrated a reduction in the phosphorylation of the α subunit of AMPK of 29% that agrees with a previously published study using the Arg302Gln PRKAG2 mice at more than 8 weeks of age [21]. With a reduction in AMPK activity, our data establish a 56% reduction in glucose uptake with FDG PET imaging in TGmut mice compared to TGwt.

As GLUT transporter efficiency is the rate-limiting step for glucose uptake and the insulin-dependent transporter GLUT4 is the most abundant transporter in the mouse myocardium, we sought to determine if FDG uptake could be increased in the PRKAG2 mutant mouse hearts following acute insulin stimulation. We found that unlike TGwt mice, where there was a 71% to 76% increase in Patlak Ki and SUV values from baseline, TGmut mice displayed no increase in FDG uptake following insulin stimulation. We additionally determined that the protein expression of the cardiac insulin receptor β is not altered between TGwt and TGmut mice. As the transgene is cardiac specific, we compared the myocardial FDG data with skeletal muscle uptake at baseline and following insulin. As expected, this alteration affects the heart only, with skeletal muscle SUV values increased similarly 31% to 38% following insulin in both genotypes. This increase correlates with previously published data in control mice with insulin pretreatment [22].

**Figure 5:**
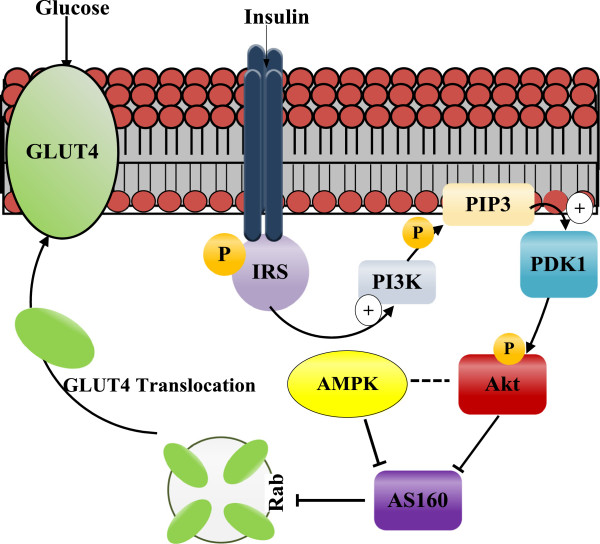
**Proposed interaction of AMPK on insulin signaling and GLUT4 translocation.** Potential targets of AMPK to alter insulin signaling of GLUT4 translocation through a direct inhibition of AS160 or via the mTOR pathway to inhibit Akt. The role that excessive glycogen levels play in the PRKAG2 mutation may also contribute to myocardial insulin resistance. PI3K, phosphatidylinositol 3-kinase; PIP3, phosphatidylinositol (3,4,5)-triphosphate; GLUT4, glucose transporter 4; PDK-1, phosphoinositide-dependent kinase-1; IRS, insulin receptor subunit.

The lack of responsiveness to cardiac insulin stimulation in the PRKAG2 mutant mice, where AMPK activity is dysfunctional, is intriguing where a link between insulin stimulation and AMPK is currently under investigation with regards to type 2 diabetes [3]. The limiting factor in our model may be the excess glycogen levels. In a normal myocardium, insulin promotes glucose uptake and glycogen synthesis [23]. Previous groups have shown that when saturated with glucose, skeletal muscles will display insulin resistance matched with a reduction in AMPK activity [24,25]. In the current study, an approximate fourfold increase in cardiac glycogen stores was observed in TGmut mice. Folmes et al. [21] reported that in adult TGmut mice, a downregulation of Akt phosphorylation and decreased AS160 phosphorylation/expression, both of which would lead to reduced glucose uptake. From this data, we would hypothesize, that the increased glycogen stores are causing a negative feedback reducing AMPK activity, and potentially altering downstream proteins in the insulin stimulated glucose pathway (Figure 5) thus reducing glucose uptake. The lack of insulin stimulation appears to agree with the theory that inhibition of AMPK activity ameliorates insulin stimulation even in the presence of glucose deprivation.

### Limitations

In this study, we used FDG as a measurement of glucose uptake. Glucose uptake can be affected by multiple factors including but not limited to GLUT and SGL transporter translocations to the cell membrane, hexokinase activity, and glucose-6-phosphate levels. We did not measure directly the sarcolemmal protein expression, mRNA or whole cell activity of these factors in this study. We recognize that free fatty acid levels affect insulin resistance and contributes to functional changes in the LV. However, in this study, plasma and tissue free fatty acid levels were not evaluated. Changes in plasma insulin levels were not measured following an acute insulin treatment, as an increase in plasma insulin values were expected.

## Conclusions

The current findings indicate that the PRKAG2 mouse model has reduced cardiac glucose uptake at 5 to 8 months of age, correlating with previous work [13] from our group with reduced uptake in affected PRKAG2 patients. This reduction in cardiac uptake occurs with reduced cardiac function, increased glycogen stores and reduced AMPK activity. Furthermore, the PRKAG2 mouse model exhibits myocardial insulin resistance. The findings of this study not only provide insight of the PRKAG2 cardiac syndrome but also emphasize the potential role of AMPK in insulin resistance observed in other pathological cardiac states. Conversely, with similarities to pathologic cardiac states such as type 2 diabetes, these findings provide further information on the role that metabolic therapeutic targets may have in the affected PRKAG2 patients.

## Competing interests

Dr Rob deKemp receives revenue shares from FlowQuant sales.

## Authors’ contributions

Genotyping, FDG imaging, echocardiography, glycogen assay, westerns, and all data analysis described in this manuscript were conducted by ST, under supervision and guidance of MG and JDS. MEH assisted with the interpretation of the biochemical assays within the context of the PRKAG2 model. RSB participated in the clinical implications and perspective on the FDG imaging data. RdK facilitated the application of kinetic modeling and quantification. All authors read and approved the final manuscript.
